# Gender differences in the provision of key post-arrest interventions for out-of-hospital cardiac arrest (OHCA) patients—protocol for a systematic review

**DOI:** 10.1186/s13643-019-1122-5

**Published:** 2019-08-13

**Authors:** Morgan Slater, Victoria M. Sparrow-Downes, Precilla Veigas, Joanna M. Bielecki, Valeria E. Rac

**Affiliations:** 1 Toronto Health Economics and Technology Assessment (THETA) Collaborative, Toronto General Hospital Research Institute, University Health Network, Toronto, ON Canada; 2 Department of Family Medicine, Queen’s University, Kingston, ON Canada; 3 Faculty of Medicine, University of Ottawa, Ottawa, ON Canada; 4 Institute of Health Policy, Management and Evaluation (IHPME), University of Toronto, Toronto, ON Canada; 5 Ted Rogers Centre for Heart Research, Peter Munk Cardiac Centre, Toronto General Hospital, University Health Network, Toronto, ON Canada

**Keywords:** Sex, Gender, Cardiac arrest, Post-arrest care, Systematic review

## Abstract

**Background:**

Evidence shows that the implementation of optimal post-arrest care significantly increases survival and functional outcomes among patients who experience an out-of-hospital cardiac arrest (OHCA). However, differences in OHCA survival have been reported between men and women, suggesting underlying differences in post-arrest care. This systematic review will evaluate gender differences in the provision of key post-arrest interventions.

**Methods:**

Eligible studies will be identified through systematic searches of relevant databases. Randomized controlled trials and observational studies of adult patients will be eligible for inclusion if they report gender-specific data on the provision of one or more guideline-based post-arrest interventions in OHCA patients who survived to hospital admission. Two independent reviewers will perform both the title and abstract and full-text screening along with data abstraction for the selected studies. Study quality will be assessed using a modified Cochrane Risk of Bias tool for RCTs or the ROBINS-I tool for observational studies. The strength of evidence for each included study will be assessed using a modified Grades of Recommendation, Assessment, Development, and Evaluation (GRADE) system.

**Discussion:**

To our knowledge, this systematic review will be the first to address the association between patient gender and the provision of post-arrest care. The findings from this systematic review will provide valuable insight to gender disparities in the provision of post-arrest care. This systematic review was designed in accordance with the Preferred Reporting Items for Systematic Reviews and Meta-Analyses (PRISMA) statement. This protocol observes the Preferred Reporting Items for Systematic Review and Meta-Analysis Protocols (PRISMA-P) statement.

**Systematic review registration:**

PROSPERO CRD42012003096

**Electronic supplementary material:**

The online version of this article (10.1186/s13643-019-1122-5) contains supplementary material, which is available to authorized users.

## Background

Cardiovascular disease (CVD) is the leading global cause of mortality, accounting for 17.9 million deaths per year in 2015, a number that is expected to reach more than 23.6 million by 2030 [1]. Although traditionally perceived to primarily affect men, recent research suggests that CVD has a pronounced effect on women’s health [2]. In fact, CVD is the primary cause of death among women and more women than men die of CVD each year [3]. Cardiac arrest remains the leading cause of CVD mortality, accounting for more than 50% of all CVD-related deaths [4]. Each year in Canada, an estimated 40,000 cardiac arrests will occur, 85% of which occur outside of a hospital setting [5]. Emergency medical services (EMS) treat 52.1 out-of-hospital cardiac arrests (OHCA) per 100,000 population; however, survival-to-discharge rates remain low [6, 7].

In the hopes of improving OHCA survival rates, the American Heart Association (AHA) International Liaison Committee of Resuscitation adopted a chain of survival concept as a guideline for the management and treatment of OHCA [8]. The chain consists of four links: need to call 911, early initiation of cardiopulmonary resuscitation (CPR), early defibrillation, and early delivery of advanced life support. In 2010, a fifth link representing early post-resuscitation care was added, emphasizing the importance of early access to quality post-resuscitation interventions including: cardiology and/or neurology consultation(s), coronary angiography, coronary artery bypass grafting (CABG), electrophysiological testing (ET), implantable cardiac defibrillator (ICD), neuroprognostication no earlier than 72 h post-arrest, percutaneous coronary intervention (PCI), and targeted temperature management (TTM) [9, 10]. Evidence has shown that implementation of optimized post-arrest care significantly contributes to increased survival and functional outcomes in OHCA patients [11].

Gender differences have been reported in OHCA survival [12–20]. A recent systematic review and meta-analysis found that overall survival among women was higher than men [12]; however, multiple studies have found that female OHCA patients are less likely to survive to hospital discharge [13, 17, 18, 20] despite being more likely to survive to hospital admission [14–17, 19]. With gender differences in the provision of care after myocardial infarction (MI) being well-established [21–25], this discrepancy in OHCA survival may be due to gender differences in post-arrest care. The objective of this systematic review is to systematically review the evidence on gender differences in the provision of post-arrest care.

## Methods

This systematic review was designed following the Preferred Reporting Items for Systematic Reviews and Meta-Analyses (PRISMA) statement [26], and this protocol follows the Preferred Reporting Items for Systematic Review and Meta-Analysis Protocols (PRISMA-P) and checklist (Additional file 1) [27, 28]. This protocol is registered with PROSPERO as CRD42012003096.

### Eligibility criteria

#### Participants

Studies must include adult (≥ 18 years of age) patients who were admitted to hospital after experiencing an OHCA and return of spontaneous circulation (ROSC). Studies including patients with a known do not resuscitate (DNR) status restricting delivery of life-saving interventions will be excluded.

#### Interventions

Based on the 2010 and 2015 AHA guidelines for post-cardiac arrest care [9, 10] and 2008 AHA consensus statement on post-cardiac arrest syndrome [29], the focus of this review will be on the provision of the following key evidence-based interventions: cardiology and/or neurology consultation(s), coronary angiography, coronary artery bypass grafting (CABG), electrophysiological testing (ET), implantable cardiac defibrillator (ICD), neuroprognostication no earlier than 72 h after arrest, percutaneous coronary intervention (PCI), and targeted temperature management (TTM) [9, 10].

#### Outcomes

Studies will be included if they report gender-specific rates of guideline-based post-arrest interventions listed above. If a study does not report gender-specific data, it will be excluded unless the authors of the study can provide either gender-stratified analyses or raw, anonymized data.

#### Study designs

Randomized controlled trials (RCTs) and observational study designs, including prospective and retrospective cohort, case-control, case series and cross-sectional studies, and published, peer-reviewed registry data, will be eligible for inclusion. Abstracts, commentaries, editorials, letters to the editor, case reports, and animal studies will be excluded.

#### Setting

Restrictions on the type of setting will not be imposed.

#### Language

Only English language publications will be included in this systematic review.

### Information sources

We will identify eligible studies through a search of the following databases: Ovid MEDLINE, Ovid EMBASE, EBSCOhost CINAHL, Cochrane Library: Cochrane Database of Systematic Reviews, Cochrane Central Register of Controlled Trials, Database of Abstracts of Reviews of Effect, and ICTRP Database. As these interventions began to be used in the 1990s, we will limit our search to publications from 1989 onward. We will also use the PROSPERO registry to identify all relevant active or completed systematic reviews. The electronic searches will be supplemented through a review of the reference lists of the eligible studies and previous systematic review.

### Search strategy

An information specialist (JB) designed and conducted the search strategy based on Cochrane review methodology by [30]. The search strategy was peer-reviewed by an independent information specialist (CZ) using the Peer Review of Electronic Search Strategies (PRESS) checklist [31]. The search includes Medical Subject Headings (MeSH) and natural language terms to capture the concepts of heart arrest, eligible interventions, and gender. A detailed search strategy for the MEDLINE (Ovid) database is provided in Additional file 2, and the final search strategy will be translated into appropriate syntax for each database.

### Data management

An EndNote library will be used to collect the results of the searches from each database; duplicate studies will be removed. Each reviewer will utilize a copy of the EndNote library to code the status of each eligible article after each stage of screening. The abstracted data from included studies will be organized and stored in a Microsoft Excel spreadsheet.

### Study selection and screening process

We will use a stepwise review process, based on the Preferred Reporting Items for Systematic Reviews and Meta-Analyses (PRISMA) [26]. Using a study eligibility form, two reviewers will independently screen the titles and abstracts of the studies identified by the search strategy and select studies for full-text review. During each stage of review, both reviewers will record the reason for exclusion. Neither reviewer will be blinded to the study authors, institutions, or journal titles.

During full-text screening, any disagreements will be resolved with the senior author (VER). We will report inter-rater agreement with a weighted kappa statistic.

### Data collection

Both reviewers will independently extract the data from the selected studies using a standard data collection form based on the Data Extraction Template for Cochrane Reviews [32]. The extracted information will be compared; any discrepancies will be resolved through discussion between the reviewers and, if necessary, the senior author. When required, we will contact the authors of eligible studies to obtain further information.

### Data items

For each included study, reviewers will extract data on the patient population (specifically, gender), study characteristics, and outcomes of interventions. Study characteristics will include the study design, study location, sample size, study period, and post-arrest intervention(s) studied, as well as the year of publication. We will capture data on the provision of any of the following evidence-based interventions: ICD, CABG, PCI, coronary angiography, TTM, ET, cardiology and/or neurology consultation(s), and/or neuroprognostication ≥ 72 hours. Both unadjusted and adjusted results will be abstracted as available.

### Risk of bias and quality assessment of individual studies

Two reviewers will independently assess bias in each study; any disagreements will be resolved through discussion with the senior author. For RCTs, we will use the Cochrane Risk of Bias tool [33]. While the Cochrane tool assesses the blinding of patients or participants, for the purpose of this review, this concept is not relevant as the interventions of interest are procedural in nature. For observational studies, we will use the Risk of Bias In Non-randomised Studies of Intervention (ROBINS-I) tool [34].

### Synthesis of results

A descriptive, narrative synthesis will be used to summarize the data from different studies. If applicable, a meta-analysis will be conducted, dependent on the number of included studies and their data homogeneity. The synthesized evidence will be framed according to the strength and quality of evidence.

### Strength of the evidence assessment

As recommended by the Cochrane Collaboration [35], two reviewers will assess the strength of evidence of the included studies using the Grades of Recommendation, Assessment, Development, and Evaluation (GRADE) approach [36]. The reviewers will independently rate the quality of each study; disagreements will be resolved through discussion with the senior author. We will also use funnel plots to assess potential publication bias [37].

## Discussion

The goal of this review is to determine if there are differences in the provision of post-arrest care between men and women. Based on existing published literature, we will analyze data extracted from studies on OHCA patients who survived to hospital admission. Study inclusion criteria include a number of key evidence-based interventions and gender stratification.

To our knowledge, this systematic review is the first to address the association between patient gender and the provision of post-arrest care. We anticipate that the results of this review may point to gaps in the published literature that specifically examines gender differences in care in this patient population.

## Additional files

Additional file 1: PRISMA checklist and flow diagram. (PDF 218 kb)

Additional file 2: Preliminary search strategy for Medline OVID. (DOCX 145 kb)

## Data Availability

Not applicable.

## Abbreviations

AHA: American Heart Association
CABG: Coronary artery bypass grafting
CDSR: Cochrane Database of Systematic Reviews
CPR: Cardiopulmonary resuscitation
CRT: Cardiac resynchronization therapy
CVD: Cardiovascular disease
EMS: Emergency medical services
ET: Electrophysiological testing
GRADE: Grades of Recommendation, Assessment, Development, and Evaluation
HF: Heart failure
ICD: Implantable cardioverter-defibrillator
PCI: Percutaneous coronary intervention
PRISMA: Preferred Reporting Items for Systematic Reviews and Meta-Analyses
PRISMA-P: Preferred Reporting Items for Systematic Reviews and Meta-Analysis Protocols
RCT: Randomized controlled trial
ROSC: Return of spontaneous circulation
SCI-EXPANDED: Science Citation Index Expanded
TTM: Targeted temperature management
