# Supplementary material for: Gender differences in the provision of key post-arrest interventions for out-of-hospital cardiac arrest (OHCA) patients—protocol for a systematic review
Source: Syst Rev. 2019 Aug 13;8:203. doi: 10.1186/s13643-019-1122-5 (PMC6692955; doi:10.1186/s13643-019-1122-5)
Supplement: Supplementary file 2 — Preliminary search strategy for Medline OVID. (DOCX 145 kb) [file 13643_2019_1122_MOESM2_ESM.docx]

**Preliminary search strategy for Medline Ovid**

| **#** | **Searches** |
| --- | --- |
| 1 | heart arrest/ or out-of-hospital cardiac arrest/ or (asystole? or ((heart or cardiac or post-cardiac or cardiopulmonary or cardio-pulmonary) adj2 arrest) or post-arrest or ((cardiac or heart) adj3 (sudden or out-of-hospital or "out of hospital") adj3 arrest) or OHCA).tw,kf. |
| 2 | exp cardiac catheterization/ or exp angioplasty balloon/ or angioplasty, laser/ or exp hypothermia, induced/ or exp defibrillators, implantable/ or exp myocardial revascularization/ or transmyocardial laser revascularization/ or circulatory arrest, deep hypothermia induced/ or angiocardiography/ or cardiography, impedance/ or coronary angiography/ or exp percutaneous coronary intervention/ or exp coronary artery bypass/ or electrophysiologic techniques, cardiac/ or neurologic examination/ or exp electrocardiography/ or diagnostic techniques, neurological/ or exp electroencephalography/ or ((catheter$ adj2 (heart or cardiac or cardiopulmonary or cardio-pulmonary)) or ((balloon or transluminal or endoluminal or laser or laser-assisted) adj3 angioplast$) or ((induced or therapeutic) adj3 hypothermia?) or ((defibrillator? or cardioverter-defibrillator?) adj3 implantable) or icd or (revasculari#ation? adj2 (myocardial or transmyocardial or trans-myocardial or laser)) or "internal mammary arter$ implantation" or (("deep hypothermic" or "deep hypothermia") adj3 circulatory adj3 arrest) or (function adj3 test? adj3 (cardiac or heart)) or "chain of survival" or angiocardiograph$ or ((cardiograph$ or transthoracic) adj3 impedance) or (coronary adj2 angiograph$) or (coronary adj4 (intervention? or revasculari#ation?) adj4 percutaneous) or (bypass$ adj4 (coronary or aortocoronary)) or ((technique? or technic? or stud?) adj2 (cardiac or intracardiac) adj2 electrophysiologic$) or (electrogram adj ((bundle adj2 his) or intracardiac or atrial)) or (programmed adj2 electrostimulation adj2 cardiac) or ((neurological or neurologic) adj prognostication) or neuroprognostication or ecg or ekg or electrocardiogra$ or (evoked adj4 potential? adj4 somatosensory) or (neurologic$ adj4 (technic? or technique?) adj4 diagnostic) or EEG or electroencephalogram? or electroencephalography? or electrocorticography? or EcoG? or "brain wave?" or brainwave? or (cortical adj4 (synchronization? or desynchronization?)) or "return of spontaneous circulation" or ROSC or "targeted temperature management" or TTM).tw,kf. |
| 3 | 1 and 2 |
| 4 | 3 not (men not women).tw. |
| 5 | 3 not (male not female).tw. |
| 6 | 3 and female/ |
| 7 | 4 or 5 or 6 |
| 8 | Animals/ not (Animals/ and Humans/) |
| 9 | 7 not 8 |
| 10 | (comment or editorial or historical article or letter or biography or festschrift or interview? or lecture? or "legal case?" or "patient education handout" or congresses).pt. |
| 11 | 9 not 10 |
| 12 | 11 not ((exp infant/ or exp child/ or adolescent/) not (exp adult/)) |
| 13 | limit 12 to yr="1990 -Current" |
